# Kolaviron Protects the Prefrontal Cortex and Hippocampus against Histomorphological and Neurobehavioural Changes in Cuprizone Model of Multiple Sclerosis

**DOI:** 10.21315/mjms2018.25.2.6

**Published:** 2018-04-27

**Authors:** Gabriel Olaiya Omotoso, Olayemi Joseph Olajide, Ismail Temitayo Gbadamosi, Mikail Abiodun Rasheed, Chiazokam Tochukwu Izuogu

**Affiliations:** 1 Department of Anatomy, Faculty of Basic Medical Sciences, College of Health Sciences, University of Ilorin, Ilorin, Nigeria; 2 International Center for Genetic Engineering and Biotechnology, Padriciano 99, Trieste-Italy

**Keywords:** animal behaviours, biflavonoids, cuprizone, demyelination, morphology

## Abstract

**Background:**

This study explored the efficacy of kolaviron—a biflavonoid complex isolated from the seeds of *Garcinia kola*—in protecting against cuprizone (CPZ)-induced demyelination in both the prefrontal cortex and the hippocampus of Wistar rats.

**Methodology:**

Thirty rats were treated to receive 0.5 mL phosphate-buffered saline (group A, control), 0.5 mL corn oil (group B), 0.2% CPZ (group C), for 6 weeks, 0.2% CPZ for 3 weeks and then 200 mg/kg of Kv for 3 weeks (group D), or 200 mg/kg of Kv for 3 weeks followed by 0.2% CPZ for 3 weeks (group E). Rats were assessed for exploratory functions and anxiety-like behaviour before being euthanised and perfused transcardially with 4% paraformaldehyde. Prefrontal and hippocampal thin sections were stained in hematoxylin and eosin and cresyl fast violet stains.

**Results:**

CPZ-induced demyelination resulted in behavioural impairment as seen by reduced exploratory activities, rearing behaviour, stretch attend posture, center square entry, and anxiogenic characteristics. Degenerative changes including pyknosis, karyorrhexis, neuronal hypertrophy, and reduced Nissl integrity were also seen. Animals treated with Kv showed significant improvement in behavioural outcomes and a comparatively normal cytoarchitectural profile.

**Conclusion:**

Kv provides protective roles against CPZ-induced neurotoxicity through prevention of ribosomal protein degradation.

## Introduction

Multiple sclerosis (MS) is the leading cause of neurological disability in young adults, with more than two million of the world population affected (1). Morbidity resulting from demyelinating diseases causes long-lasting disability that impedes sufferers from leading normal lives. In MS, structurally distinct pathological alterations are observed in both white and gray matter, leading to various forms of neurological dysfunctions (2, 3). Previous studies have reported the presence of demyelination in the hippocampus formation of individuals with MS, and the degree of this lesion strongly correlates with progression of cognitive dysfunctions seen in such patients (4). Cases of neurodegenerative diseases are generally under-reported in developing countries; however, there is increased evidence of a rise in the prevalence of MS among patients with neurological disorders. Despite the severity of the problem, the few therapies that attempt to retard the progression of demyelination/neuronal loss in MS are largely ineffective. Therefore, evolving therapeutic targets aimed at alleviating or managing such neurodegenerative conditions as MS are noteworthy, and considerable global attention is drawn toward developments in this area. Cuprizone (CPZ) is a copper chelator that has widely been used as an agent to induce demyelination of the central nervous system in order to study cellular events involved in demyelination and remyelination (5). On the other hand, kolaviron (Kv) is a natural anti-oxidant and anti-inflammatory bioflavonoid complex isolated from the seeds of *Garcinia kola*—an oral masticatory nut cultivated in West Africa (6). Kv has a well-defined structure and an array of biological activities including anti-oxidant, anti-diabetic, anti-genotoxic, and hepatoprotective properties, and its role in reducing the production of reactive oxygen species has been reported (7). Although the beneficial properties of Kv as an anti-oxidant and anti-inflammatory agent in the biological system have been documented in several studies involving in vivo and in vitro disease models (8–10), its therapeutic potentials against degenerative changes associated with demyelination and neurotoxicity are unknown. Therefore, this study aimed to determine the beneficial roles of Kv against neurobehavioural alterations associated with CPZ-induced demyelination of the central nervous system and to elucidate Kv inhibitory properties on the concurrent neuropathological damage in both the prefrontal cortex (PFC) and the hippocampus of rats.

## Materials and Methods

### Experimental Animals

Inbred adult male Wistar rats were nurtured at the animal-holding facility of the Faculty of Basic Medical Sciences, University of Ilorin, Nigeria. The rats were kept in clean wire gauze cages where they were served rat chow and water ad libitum. All protocols and treatment procedures complied strictly with the Institutional Animal Care and Use Committee (IACUC) guidelines as approved by the College of Health Sciences Ethical Committee, University of Ilorin, Nigeria.

### Garcinia kola and Cuprizone Procurement

Seeds of *Garcinia kola* (5 kg) were obtained from a single source at a market in Ilorin, north central part of Nigeria. The seeds were then authenticated at the Department of Plant Biology, Faculty of Life Sciences, University of Ilorin, Nigeria. It was verified that the seeds were of uniform species, and the specimen voucher number in the herbarium was UILH/001/1217. Cuprizone was purchased from Sigma-Aldrich, Germany. Petroleum ether, acetone, and ethyl acetate were products of Thermo Fisher, USA, purchased from Laboratory Trae Scientific Chemical Store in Ilorin, Nigeria. All other reagents were sourced within the lab and were of bioscience grade.

### Isolation and Purification of Kolaviron from Garcinia kola

*Garcinia kola* seeds were air-dried at room temperature (28 °C–30 °C) for 3 weeks. Subsequently, Kv was isolated from the dry seeds of the kola and characterised according to previously described methods (11, 12). The following describes the process briefly: Powdered seeds (1.87 kg) were extracted with light petroleum ether (BP 40 °C–60 °C) in a Soxhlet extractor for about 3 h per round. The defatted, dried marc was repacked and then extracted with acetone. The extract was concentrated and diluted to twice its volume with distilled water and extracted with ethyl acetate (4.5 L). The concentrated ethyl acetate fraction gave a yellow solid known as Kv. The purity and identity of Kv was determined by subjecting it to thin-layer chromatography using silica-gel-GF254-coated plates and a solvent mixture of methanol and chloroform in a ratio 1:4 v/v. The separation revealed the presence of 3 bands, which were viewed under UV light at a wavelength of 254 nm with RF (ratio to front) values of 0.48, 0.71, and 0.76. The yield of the preparation was 5.5%. A dose of 200 mg/kg of Kv was used (12).

### Preparation of Other Treatment Solutions

Phosphate-buffered saline (PBS; 0.1 M) was freshly prepared. Kv was dissolved in corn oil (Carlini, ALDI Inc., Batavia) (80 mg/mL), which served as vehicle for oral treatment. CPZ (2 g) was made up to 1000 g with standardised rat chow to make a 0.2% CPZ concentration for oral administration (5). Administration of Kv to rats across all groups was done using a modified oral cannula.

### Treatment of Experimental Animals

Thirty Wistar rats were randomly distributed into five groups (A–E) of six animals each:

1. Group A: Control; given 0.5 mL PBS for 6 weeks, p.o.
2. Group B: Untreated; given 0.5 mL of corn oil as a vehicle for Kv administration, for 6 weeks, p.o.
3. Group C: 0.2% CPZ in standard diet for 6 weeks, p.o.
4. Group D: 0.2% CPZ in standard diet for 3 weeks followed by 200 mg/kg Kv for another 3 weeks, p.o.
5. Group E: 200 mg/kg Kv for 3 weeks and then 0.2% CPZ in standard diet for another 3 weeks, p.o.

### Neurobehavioural Studies

#### Elevated Plus Maze

Differences in the anxiety levels of rats were evaluated across treatment groups using this method (13). The rats were introduced into an elevated plus apparatus that stood 45 cm tall with two open arms and two closed arms for an exploration time of 5 min. The open-arm duration (OAD) was estimated as the total time the rats spent in the open arm of the elevated plus maze for the duration of the study.

#### Open Field Test (OFT)

The open field apparatus was made from plywood measuring 100 cm × 100 cm with walls that were 50 cm high (14). With a blue marker, the floor was divided into square grids each measuring 25 cm in length; a red marker was used to draw a center square of the same length. During the test, the rats were picked up by their tails, dropped in the center square, and allowed to explore for 5 min while a video recorder, which was placed above the apparatus, captured their activities. Five behavioural parameters were scored from the OFT: the number of lines crossed, center square entry, center square duration, rearing frequency, and stretch attend posture. The number of lines crossed was the frequency with which the rats had all four paws cross one of the grid lines. The center square entry was the frequency with which the rats had all four paws cross one of the red lines into the central square, whereas the center square duration was the total time spent in the center square. The rearing frequency was the number of times the animal stood on its hind limbs. The stretch attend postures were the frequency with which the animal demonstrated forward elongation of the head and shoulders followed by retraction to the original position.

### Tissue Processing

A day after last administration, rats were euthanised using 20 mg/kg of ketamine (intramuscular) and subjected to transcardial perfusion in which a flush of 50 mL of 0.1 M PBS (pH 7.4) was followed by 500 mL of 4% paraformaldehyde (PFA). The brain tissues were then excised and post-fixed in 4% PFA for 24 h before being subjected to processing. The PFCs and hippocampi were excised; afterward, the sections were processed routinely to obtain paraffin-wax-embedded blocks for histology and histochemistry. Histological demonstration of cortical and hippocampal cytoarchitecture was carried out in paraffin-wax-embedded sections, which were stained in hematoxylin and eosin, while Nissl substances were demonstrated with cresyl fast violet stain.

### Data Analysis

All quantitative data were analysed using the GraphPad Prism® software (version 6). Neurobehavioural outcomes were plotted in ANOVA with Tukey’s multiple comparisons test. Significance was set at *P* < 0.05 (95% confidence interval). The outcomes were represented in bar charts with error bars to show the mean and standard error of the mean.

## Results

### Weight Observations

**Figure 1:**
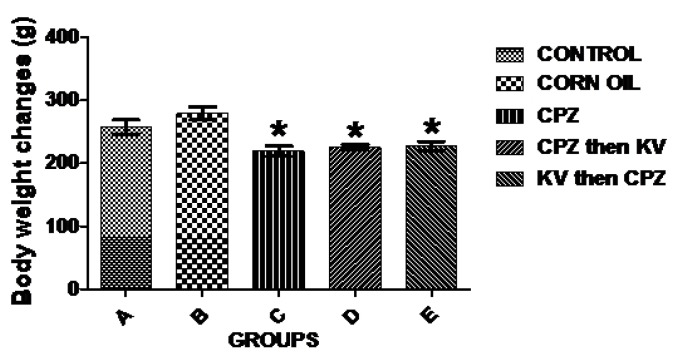
Analysis of the body weight changes of rats in the experimental groups. CPZ-treated rats with or without Kv (C, D and E) showed significantly reduced body weight compared to the control (A). Slight increase was also notable in corn oil-treated rats. * is significant difference compared to control group at *P* < 0.05. CPZ = cuprizone; KV (or Kv) = kolaviron

**Figure 2:**
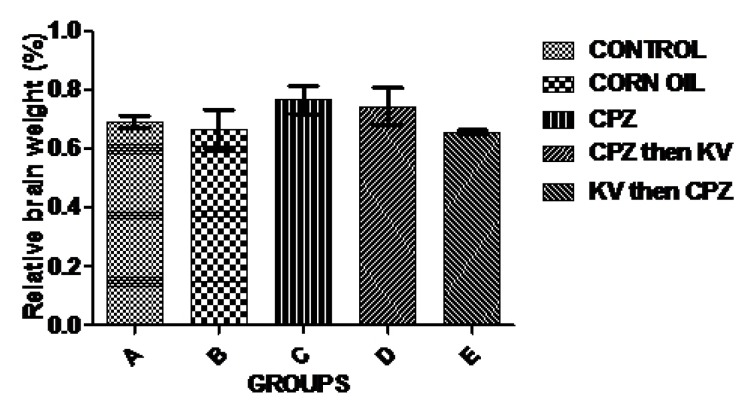
Relative brain weight of animals in the experimental groups, showing no statistically significant changes. CPZ = cuprizone; KV (or Kv) = kolaviron

Evaluations of body weight changes and relative brain weights were analysed across the different treatment groups (Figure 1). When compared to the control (A), the body weights of rats treated with CPZ with or without Kv in groups C, D, and E were significantly reduced. Rats in group B (corn oil) showed a slight increase in body weight when compared to the control group, which was, however, not statistically significant. The data extrapolated from the analysis of the relative brain weight, which was estimated as the ratio of brain weight to body weight, showed no significant difference across the experimental groups (Figure 2).

### Neurobehavioural Observations

**Figure 3:**
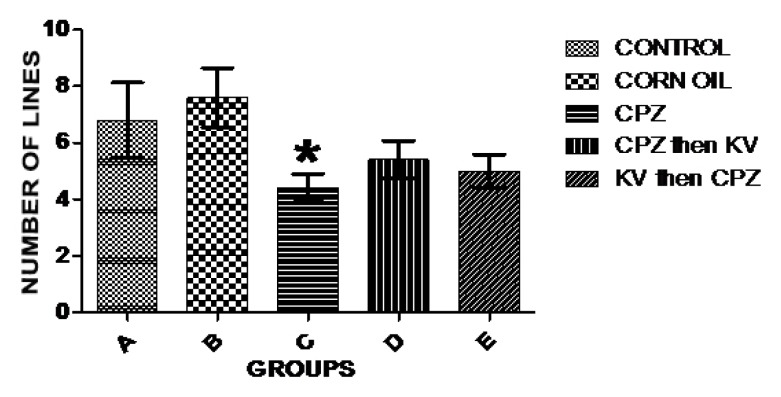
Analysis of the number of lines crossed in the open field across experimental groups. There was significant decrease in exploratory activities of the CPZ-treated group (C) compared to control (A). * is significantly different compared to control group at *P* < 0.05. CPZ = cuprizone; KV (or Kv) = kolaviron

**Figure 4:**
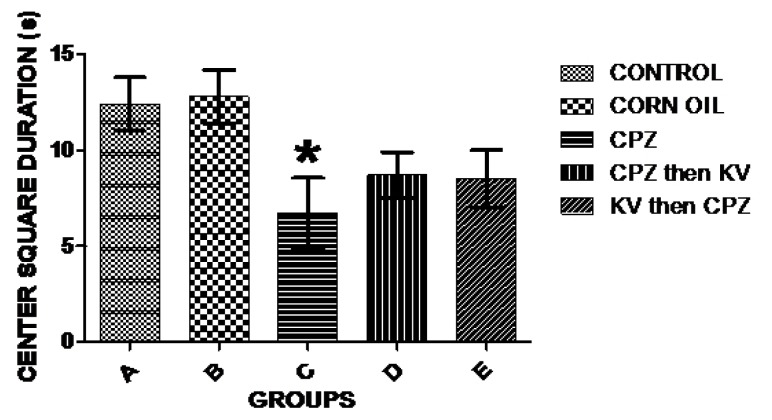
Analysis of time spent by rats at the center square of the open field across experimental groups. CPZ-treated rats (C) spent a lesser time in the open field which is significantly different (*P < 0.05) compared to the control (A). CPZ = cuprizone; KV (or Kv) = kolaviron

**Figure 5:**
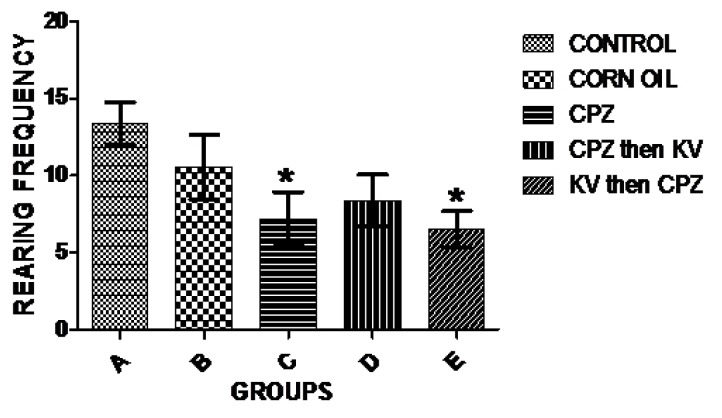
Analysis of the rearing frequency of rats in the open field showing that rats treated with CPZ (C) and Kv then CPZ (E) have significantly decreased rearing behaviour compared to control (A). Rats that received Kv intervention after CPZ administration (D) were not different from control. *significant difference compared to control (*P* < 0.05). CPZ = cuprizone; KV (or Kv) = kolaviron

Exploratory activities of rats were examined in OFT—a common measure of exploratory behaviour and general activity in rats. Analysis of the number of lines crossed in the open field was used to measure the explorative drive of rats after the different treatments. Figure 3 shows there was a significant decrease in exploratory activities of rats treated with CPZ (C) compared to control rats (A). However, Kv treatment after or before CPZ administration (D and E, respectively) improved the exploratory drive of rats to levels seen in the control. Subsequent analysis of the duration of time the rats spent in the central square of the open field (Figure 4) showed that CPZ-treated rats (C) spent significantly less time in the open field compared to the control (A), suggesting that CPZ induced anxiety and poor motor functions in rats. However, time spent in the central square by rats in the groups treated with Kv after or before CPZ administration was not significantly different from the control, indicating beneficial roles of Kv in preventing motor dysfunctions in rats. Also, analysis of the rearing frequency of rats in the open field (Figure 5) showed that rats treated with CPZ (C) and Kv followed by CPZ (E) have significantly decreased rearing behaviour, indicating high levels of anxiety compared to the control (A). However, rats that received Kv intervention after CPZ administration (D) showed normal levels of anxiety in the open field, shown by rearing frequency, which was not significantly different from the control.

**Figure 6:**
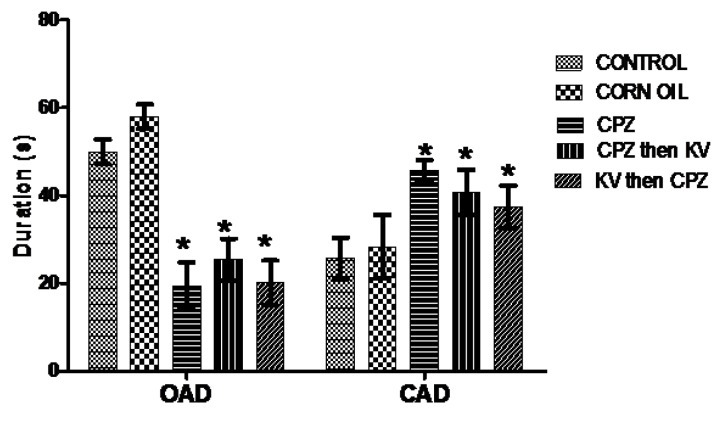
Elevated plus maze test showing analyses of the open arm duration (OAD) and closed arm duration (CAD) of rats. CPZ-treated rats with or without Kv intervention spent significantly lesser time (*P < 0.05) in OAD, and significantly higher time (*P < 0.05) in CAD than the control rats. CPZ = cuprizone; KV (or Kv) = kolaviron

**Figure 7:**
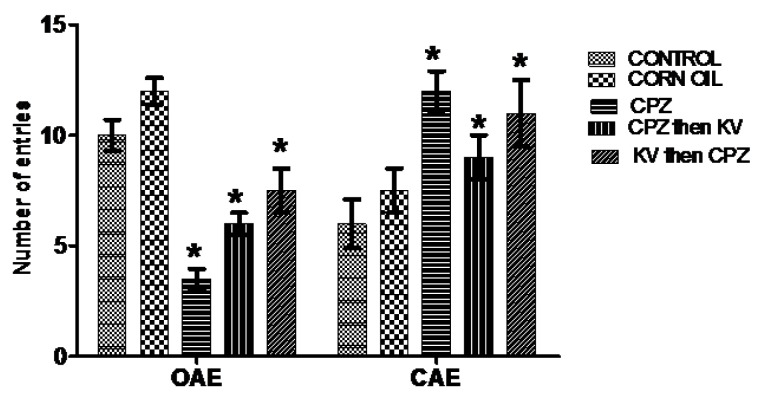
Elevated plus maze test showing analyses of the open arm entry (OAE) and closed arm entry (CAE) of rats. OAE: CPZ only and CPZ with Kv rats scored a significantly lower entry frequency than the control, while rats which received Kv before CPZ had similar scores with control and corn oil groups. CAE: Rats treated with CPZ with or without Kv intervention showed significantly higher entry frequency to the closed arm of the maze than the control rats (*P < 0.05). CPZ=cuprizone; KV (or Kv)= kolaviron

**Figure 8:**
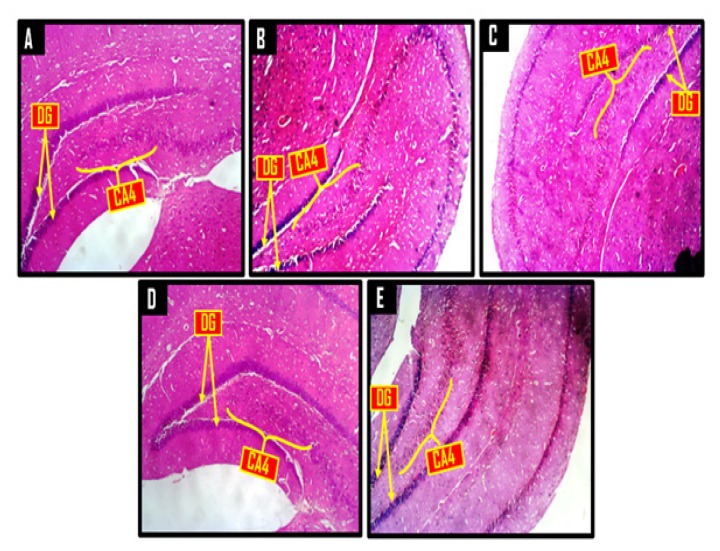
Representative photomicrographs of a panoramic view of the hippocampus showing the dentate gyrus (DG) encapsulating the conus amonis 4 (CA4) region. A = control, B = corn oil (CO), C = cuprizone (CPZ), D = cuprizone then kolaviron (CPZ then Kv) and E = kolaviron then cuprizone (Kv then CPZ). H & E ×40

Anxiolytic and anti-panic potentials of Kv in inhibiting anxiogenic cascades generated by CPZ treatment in rats were investigated in the elevated plus maze. Analysis of the OAD of rats in the elevated plus maze test (Figure 6) showed that corn oil treatment (B) did not alter the OAD compared to the control (A), while all CPZ-treated rats with or without Kv intervention (C, D, and E) spent significantly less time in the open arm of the maze than did the control rats (A). Furthermore, analysis of the closed-arm duration (CAD) of rats in the elevated plus maze (Figure 6) showed that all CPZ-treated rats with or without Kv intervention (C, D, and E) spent a significantly higher duration in the closed arm of the maze when compared to the control rats (A). Analysis of open-arm entry (OAE) frequency of rats in the elevated plus maze (Figure 7) showed that groups of rats treated with CPZ only (C) and CPZ followed by Kv (D) scored a significantly lower entry frequency than did the control rats (A). However, the OAE frequency of rats treated with Kv followed by CPZ (E) was similar to groups A and B, suggesting the therapeutic roles of Kv. Figure 8 shows the analysis of closed-arm entry (CAE) in the elevated plus maze across treatment groups. Corn oil treatment (B) did not alter the CAE frequency of rats compared to the control; however, rats treated with CPZ with or without Kv intervention (C, D, and E) showed a significantly higher entry frequency to the closed arm of the maze than did the control rats.

### Histological and Histochemical Observations

**Figure 9:**
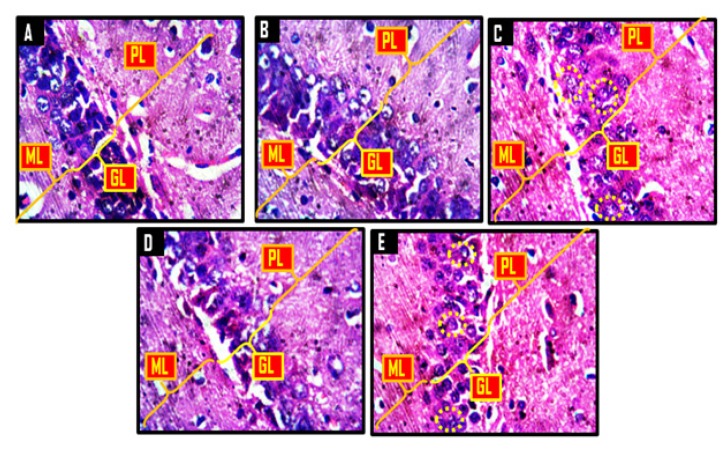
Representative photomicrographs showing molecular layer (ML), granular layer (GL) and polymorphic layer (PL) of the dentate gyrus. A = control, B = corn oil (CO), C = cuprizone (CPZ), D = cuprizone then kolaviron (CPZ then Kv) and E = kolaviron then cuprizone (Kv then CPZ). A and B showed dense and compacted granule cells in the granular layer, while the granule cells in D was less dense than what was obtainable in A and B. C and E showed apoptotic granule cells (yellow dotted circles). H & E ×400

**Figure 10:**
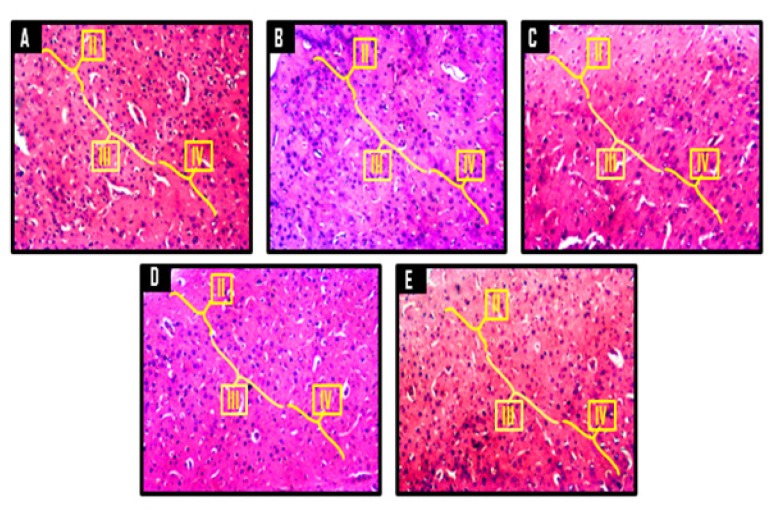
Representative photomicrographs of the dorsomedial prefrontal cortex of Wistar rats showing the external granular layer (II), external pyramidal layer (III) and the internal granular layer (IV). A = control, B = corn oil (CO), C = cuprizone (CPZ), D = cuprizone then kolaviron (CPZ then Kv) and E = kolaviron then cuprizone (Kv then CPZ). A and B showed normal cortical delineation and cellular density while C showed poor cortical delineation with reduced cellular density. D and E showed typical cortical delineation but reduced cellular density relative to A and B. H & E ×100

**Figure 11:**
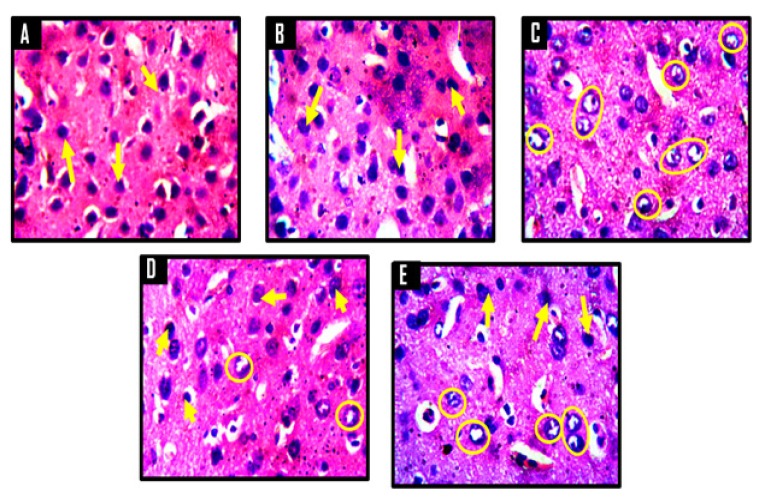
Representative photomicrographs of the internal granular layer of the prefrontal cortex of Wistar rats. A = control, B = corn oil (CO), C = cuprizone (CPZ), D = cuprizone then kolaviron (CPZ then Kv) and E = kolaviron then cuprizone (Kv then CPZ). A and B showed deeply stained nuclei of neuronal and non-neuronal cells (yellow arrows) within their respective neuropils, while C showed numerous apoptotic cells indicated by poorly stained nuclei (yellow circles). D and E showed few apoptotic cells (yellow circles) relative to C and fewer normal neuronal and non-neuronal cells (yellow arrows) relative to A and B. H & E ×400

A panoramic view of the hippocampal morphology in the brains of rats treated with PBS and corn oil (CO) (A and B, respectively; Figure 8) has properly delineated dentate gyri with a regular density of granule cells. Cellular clusters with pyknotic characteristics were observed in the CA4 region of the hippocampi of groups C (CPZ) and D (CPZ then KV). On the other hand, the hippocampal morphology of rats in group E appeared normal, with evenly stained nuclei, which had close similarities with those observed in groups A and B. At higher-power magnification (Figure 9), the detailed nature of the cellular components of the granular layers of the dentate gyri in A and B comprised compactly packed granule cells with normally stained nuclei. However, the CPZ-treated rats showed degenerative changes mainly characterised by poorly stained granule cells in the granular layer of the dentate gyrus. The degenerative changes, however, were reversed in group D and prevented in group E, as granule neurons in both groups were succinctly demonstrated and similar to the control. Figure 10 shows a panoramic view of the PFC across treatment groups. In the CPZ-treated rats (C), the general morphology of the PFC was characterised by fragmentations of neuropil, chromatolytic, and pyknotic changes around the pyramidal and granule cells. It was seen, however, that the PFC morphology in groups D and E had typical cortical delineation and cellular density, which was similar to what could be seen in groups A and B. Similarly, Figure 11 shows that the morphology of neurons within the pyramidal and granular layers of the PFC was extensively stained, with no indication of apoptosis. Again, the neuronal cells in the PFC of CPZ-treated rats showed numerous apoptotic changes within the cortical layers. Similar to findings from the hippocampal cytoarchitectural demonstration, the extent of cellular damage in the PFC of rats in groups D and E was reversed and prevented, respectively. Neuronal morphology in both groups was normal and similar to that observed in the control.

**Figure 12:**
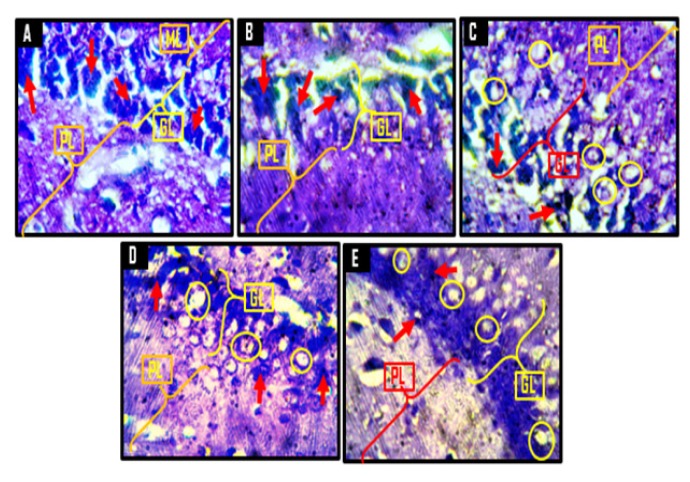
Representative photomicrograpghs of the granular layer (GL) and polymorphic layer (PL) of the dentate gyrus of the hippocampus showing the Nissl substances. A = control, B = corn oil (CO), C = cuprizone (CPZ), D = cuprizone then kolaviron (CPZ then Kv) and E = kolaviron then cuprizone (Kv then CPZ). A and B showed deeply stained granule cells (red arrows) of the granular layer with no chromatolytic cells. C revealed numerous chromatolytic granule cells (yellow circles) with very few adequately stained granule cells. In D and E, there were few chromatolytic cells in the granular layer (yellow circles) with few properly stained Nissl substances (red arrows) relative to A and B. Cresyl Fast Violet Stain ×400

**Figure 13:**
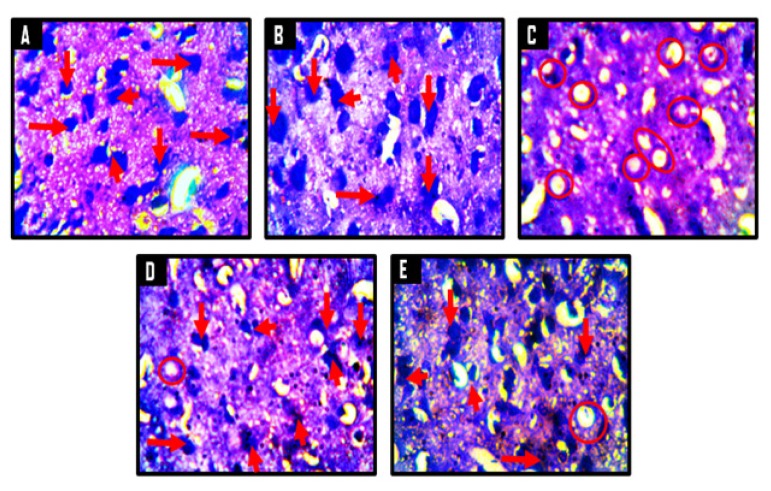
Representative photomicrographs of the internal molecular layer of the prefrontal cortices of Wistar rats showing the Nissl substances in the neuronal cells. A = control, B = corn oil (CO), C = cuprizone (CPZ), D = cuprizone then kolaviron (CPZ then Kv) and E = kolaviron then cuprizone (Kv then CPZ). A and B showed abundant Nissl substances within the neurons (red arrows), while C showed severe chromatolysis as indicated by poorly stained Nissl substances in the neurons (red circles). D and E presented with mild chromatolysis relative to C. Cresyl Fast Violet Stain ×400

A demonstration of Nissl proteins using cresyl violet staining methods across hippocampal and PFC sections (Figures 12 and 13) revealed normal Nissl profiles within the perikarya of groups A and B compared to group C, which was characterised by sparse Nissl distribution. In line with earlier findings, however, Kv treatment markedly improved the distribution and population of Nissl bodies within hippocampal and PFC neurons of rats in groups D and E. Summarily, histological and histochemical analysis of cortico-hippocampal sections of rats revealed that Kv administration provides protective roles against CPZ-induced neuronal death through a mechanism that involves prevention of ribosomal protein degradation.

## Discussion

The rationale of novel therapeutic targets in disease conditions is aimed at slowing the rate of degenerative processes and thus the symptomatic outcomes (15). The present study assessed the novelty of Kv in inhibiting CPZ-induced behavioural deficits and the associative histological and histochemical changes within the PFC and hippocampus. Some research models have noted the cytoprotective properties of Kv, including its neuroprotective roles in different models of neurological disorders (16–18), and a possible mechanism of action has been suggested (7, 16–18).

Evaluation of the body weights of rats across the treatment groups showed that CPZ administration caused significant weight loss compared to the control, which was not improved by pre- or post-treatment with Kv. In line with this finding, an earlier study (19) also found that mice on a CPZ diet lost weight at the end of a 6-week treatment period when compared to the control. The inability of Kv to reverse weight loss in CPZ-treated rats is quite intriguing, as previous reports have shown that it restores appetite in rats, which leads to subsequent weight gain (6). However, the current result may be explained by the lateness of Kv in promoting functional recovery against CPZ-induced neurotoxicity and metabolic impairment. It was also notable that the relative brain weights of rats were not significantly different across treatment groups.

Furthermore, it was shown in the open field that CPZ administration significantly reduced exploratory activities (number of lines crossed) and caused anxiety and emotional imbalance (rearing behaviour and time spent in central square, respectively) when compared to the control. A study similarly found that mice treated with 0.2% CPZ formulation showed behavioural alterations compared to the control in terms of the distance traveled in the center area, the number of defecations, and the number of rearings in an open field (20). In the present study, subsequent analysis of behavioural outcomes in the open field showed that administration of Kv to rats before or after CPZ-induced demyelination reversed or prevented loss of exploratory drive, emotional imbalance, and anxiety. The exact mechanisms potentiating CPZ-induced demyelination remain elusive; however, it is believed to involve oxidative impairment from unrelenting mitochondrial stress brought on by the toxicity of CPZ itself and effector functions of an innate immune response (21). Kv, on the other hand, has been shown to possess the major ability to scavenge free radicals resulting from oxidative impairment (22) and to halt inflammatory responses in cells (10). It is suggested, therefore, that Kv improved behavioural outcomes in this study by exerting its anti-oxidant properties in neural cells, thereby halting other excitotoxic stimuli that may result in behavioural deficits.

The elevated plus maze is a recognised and acceptable technique of assessing anxiety-related behaviour in rodents (23, 24). Considering results from the elevated plus maze, analysis of the OAD of rats showed that CPZ administration with or without Kv intervention induced anxiogenic characteristics in rats, as seen by the significantly less amount of time spent in the open arm of the maze compared to the control. CAD analysis further confirmed the anxiogenic properties of CPZ in rats. Surprisingly, Kv treatment neither before nor after CPZ administration showed significant behavioural improvement in rats in the maze. When factored in, the OAE frequency of rats in the elevated plus maze further revealed that CPZ administration causes fear and panic in rats, shown by a significantly lower entry frequency compared to the control. Notably, the OAE frequency of rats treated with Kv followed by CPZ was comparable to the levels in the control, indicating the anti-fear and anti-panic potentials of Kv.

Histological findings in this study support reports that CPZ extensively destroys hippocampal neurons (25, 26) and induces neurotoxicity in the brain, as indicated by the observed cellular clusters with pyknotic characteristics, which were observed in the PFC layers, the CA4 region of the hippocampus, and the poorly stained granule cells in the granular layer of the dentate gyrus of groups treated with CPZ. Further histological analysis suggests that treatment of rats with Kv prevented CPZ-induced neuronal degeneration, especially when given before CPZ rather than after CPZ. The neuroprotection afforded by Kv on PFC and hippocampal neurons may be linked to its previously reported anti-oxidant properties (16, 17, 27). In addition, CPZ treatment markedly reduced Nissl bodies within the PFC and hippocampus when compared to the control. Nissl bodies are essential to the well-being of a neuron, and a decrease of Nissl bodies in a neuron indicates neural degeneration (28). Therefore, the shown anti-proteolytic potentials of Kv in preventing Nissl degeneration probably underlie its therapeutic mechanisms in improving behavioural and cellular functions in CPZ-treated rats, a role that could be aligned with its strong anti-oxidant properties.

## Conclusion

The results of this study suggest that Kv has therapeutic potentials against degenerative changes associated with demyelination and neurotoxicity. It has recently been shown that agents with the potential to reverse the processes of demyelination in neurons can be used in the treatment of MS. Therefore, further studies in line with our findings may provide useful insights that will enable the development of Kv into an interventional molecule for treatment of MS.
